# Positive or negative allosteric modulation of metabotropic glutamate receptor 5 (mGluR5) does not alter expression of behavioral sensitization to methamphetamine

**DOI:** 10.12688/f1000research.2-84.v1

**Published:** 2013-03-12

**Authors:** Peter R Kufahl, Natali E Nemirovsky, Lucas R Watterson, Nicholas Zautra, M Foster Olive

**Affiliations:** 1 Department of Psychology, Arizona State University, Tempe, AZ, 85287-1104, USA

## Abstract

We investigated the role of metabotropic glutamate receptor type 5 (mGluR5) in methamphetamine-induced behavioral sensitization. The mGluR5 positive allosteric modulator (3-cyano-N-(1,3-diphenyl-1H-pyrazol-5-yl) benzamide (CDPPB) and negative allosteric modulator fenobam were tested in separate experiments. Sprague-Dawley rats were repeatedly injected with 1 mg/kg methamphetamine or saline, and then given a locomotor challenge test using a dose of 0.5 mg/kg methamphetamine. Prior to the challenge test session, rats were injected with CDPPB, fenobam, or a vehicle.  Doses from previous studies showed reduced drug-conditioned behavior; however in this study neither CDPPB nor fenobam pretreatment resulted in an altered expression of behavioral sensitization, indicating a lack of mGluR5 involvement in sensitized methamphetamine-induced locomotion. Additionally, the high dose (30 mg/kg) of fenobam resulted in decreased methamphetamine-induced locomotion in rats regardless of drug exposure history, which suggests evidence of nonspecific behavioral inhibition.

## Introduction

Compulsive drug use and associated maladaptive behaviors are cardinal features of methamphetamine (METH) addiction, and have been strongly associated with the neurochemical consequences of repeated METH abuse ^1– 3^. Among the various neurotransmitter systems affected by METH exposure is the glutamate system, where long-lasting drug-induced changes are suspected factors underlying craving and persistent vulnerability to relapse ^4^. Due to their dual roles in mediating glutamatergic synaptic plasticity and control of synaptic glutamate release, the metabotropic glutamate receptors (mGluRs) have emerged as therapeutic targets of interest in the study of drug addiction ^5^. Antagonizing the excitatory postsynaptic metabotropic glutamate receptor 5 (mGluR5) has been recently shown to attenuate the reinforcing effects of METH on a progressive ratio schedule, as well as attenuating drug-seeking behavior in rats previously trained to self-administer METH ^6^. Selective stimulation of mGluR5 has been found to improve the rate of extinction learning in rats previously conditioned to the reinforcing effects of cocaine. This study investigated the role of mGluR5 in the behavioral changes induced by repeated exposure to METH, using positive and negative allosteric modulators of mGluR5 function in separate experiments.

The consequences of chronic METH abuse are often studied in the rat model of behavioral sensitization, where chronic METH injections reliably induce an elevated locomotor response to a subsequent METH challenge, relative to rats with no prior history of METH exposure ^8– 11^. Through their interactions with the dopaminergic projections of the medial forebrain, mGluRs have been found to have roles in both the development and expression of psychostimulant sensitization ^12^. mGluR5 has been associated with the locomotor response and reinforcement attributes of psychostimulants since mice lacking this receptor were found not to respond to or self-administer cocaine as wild-type mice ^13^. While antagonism of group I mGluRs, which includes mGluR5, in subsequent experiments has generally failed to convincingly affect locomotor sensitization to cocaine ^14^, the effects of positive allosteric modulation on psychostimulant sensitization have so far remained untested. We evaluated the effect of the mGluR5 positive allosteric modulator (PAM) 3-cyano-N-(1,3-diphenyl-1H-pyrazol-5-yl)benzamide (CDPPB) and the mGluR5 negative allosteric modulator (NAM) fenobam on the expression of behavioral sensitization to METH. We utilized doses of CDPPB that have been shown to improve extinction learning after METH [30 mg/kg ^15^], and cocaine [60 mg/kg ^7^], self-administration training, and doses of fenobam (10–30 mg/kg) that have effectively reduced drug-seeking in METH-trained rats in our laboratory ^16^.

## Methods and materials

### Subjects

Eighty-eight male Sprague-Dawley rats (Harlan Laboratories, Livermore, CA), weighing 250–275 g, were pair-housed on arrival in a humidity-controlled colony room and maintained in a reversed light/dark cycle with free access to food and water throughout the experiment. All experimentation was conducted during the dark phase of the light/dark cycle. All procedures were conducted with the approval of the Institutional Care and Use Committee at Arizona State University and in accordance with the principles of the Guide for the Care and Use of Laboratory Animals (National Research Council) ^17^.

### Drugs

3-cyano-N-(1,3-diphenyl-1H-pyrazol-5-yl)benzamide (CDPPB, custom synthesized by Chemir Analytical Services, Maryland Heights, MO) was suspended in 10% *v/v* Tween 80 via sonication to form a 60 mg/ml concentration for intraperitoneal (i.p.) administration. Fenobam (1-(3-chlorophenyl)-3-3-methyl-5-oxo-4H-imidazol-2-yl) urea (custom synthesized by Chemir Analytical Services) was suspended in 0.3% *v/v* Tween 80 vehicle to form a 30 mg/ml concentration for i.p. administration. (+)Methamphetamine hydrochloride (Sigma Aldrich, St Louis, MO) was dissolved in sterile saline for i.p. administration.

### Locomotor testing procedures

**Figure 1.**
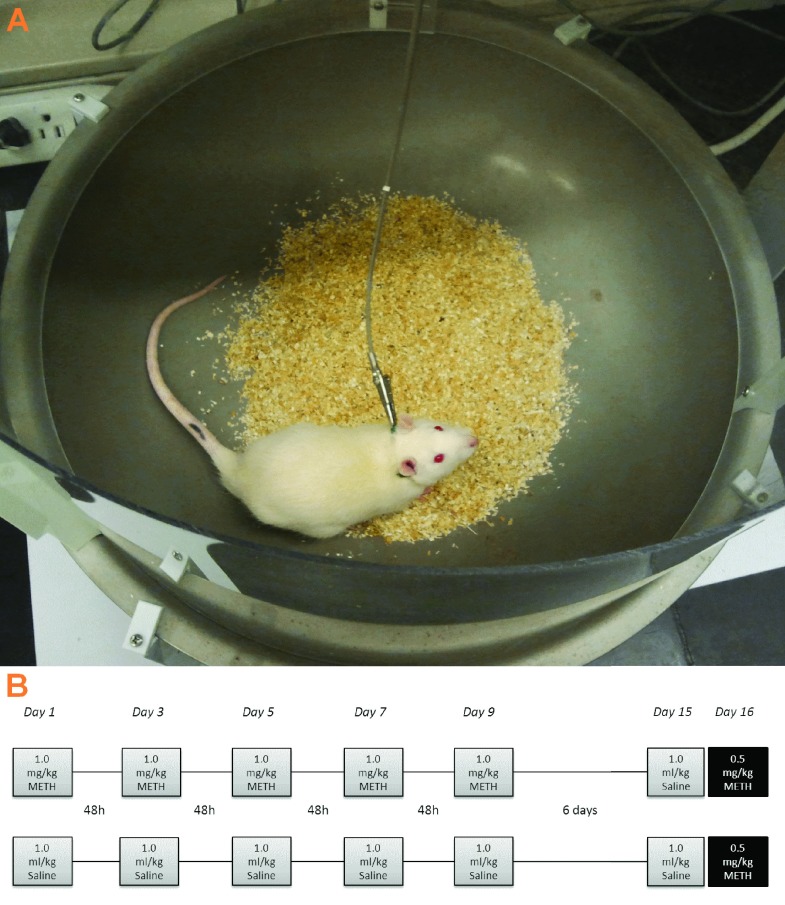
Apparatus and experimental protocol. The locomotor apparatus ( **A**) consists of a rotating actuator anchored to a U-shaped bracket over a steel bowl-shaped arena (Med Associates; 18 in top diameter, 6 in bottom diameter, 6 in depth) containing a layer of Sani-chip bedding. The rat is attached to the actuator via 45 cm spring leash terminated with an alligator clip, which is hooked onto a cable tie around the neck for the duration of the test session. The apparatus registers rotational movements as the rat causes the actuator to pivot, accumulated by computer as quarter turns. The experimental procedure ( **B**) consisted of three days of acclimation sessions in the locomotor arenas, followed by five injections of METH (1.0 mg/kg, i.p.) or saline separated by 48 hr (Days 1, 3, 5, 7 and 9). After each injection, rats were placed into the locomotor arenas for 90 min and their rotational data were recorded as quarter turns. Rats underwent locomotor testing following a saline injection on Day 15, and these data were balanced between groups assigned to mGluR5 treatment or vehicle treatment. On Day 16, the rats were given an injection of the mGluR5 ligand (CDPPB or fenobam) or vehicle, and tested 30 min later following a probe injection of METH (0.5 mg/kg, i.p.).

Locomotor activity was assessed in a Rotorat System apparatus (Med Associates, Mt. St Albans, VT) that measured rotational ambulation, quantified as quarter turns in both directions, within a bowl-shaped arena ( Figure 1A). The rats ( *N*=43 in the CDPPB experiment, *N*=45 in the fenobam experiment) were divided into groups where half of the rats received five injections of 1 mg/kg METH dissolved in saline (1 ml/kg, i.p.), separated by 48 hours, and the other half received injections of saline of matching volume ( Figure 1B). Each injection was immediately followed by a 90 min locomotor test session. After a 6-day waiting period in the colony room, all rats were given a saline injection (1 ml/kg, i.p.) and subjected to a locomotor test session. The next day, rats were injected with 0 ( *N*=7), 30 ( *N*=8) or 60 mg/kg ( *N*=6–7) CDPPB in one experiment; or 0 ( *N*=8), 10 ( *N*=8) or 30 mg/kg ( *N*=6–7) fenobam in the other experiment, and 30 min later given a challenge dose of 0.5 mg/kg METH and subjected to a 90 min locomotor test session.

Additional experiments were conducted to examine the effects of mGluR5 modulation on baseline locomotion. Rats were acclimated to the apparatus in 90 min sessions for two consecutive days, and on the next day given a 90 min locomotor test session 30 min after treatment with 0, 30 or 60 mg/kg CDPPB in one experiment ( *N*=5); or 0, 10 or 30 mg/kg fenobam in another experiment ( *N*=5).

### Data analysis

Data analysis procedures were performed using Prism 5 (GraphPad, La Jolla, CA). For the sensitization experiments, quarter turn data (in either direction, totaled over 90 min) taken during the five chronic treatment sessions were analyzed using 2-way ANOVA with *METH history* (naïve, METH-treated) as a between-subjects factor and *day* (1, 3, 5, 7 or 9) as a within-subjects factor. Locomotor behavior exhibited during the challenge sessions were quantified as quarter turns and analyzed using 2-way ANOVA with *METH history* and *treatment* (0, 30 or 60 mg/kg for the CDPPB experiment, and 0, 15 or 30 mg/kg for the fenobam experiment) as between-subjects factors. Significant interaction effects were followed by pairwise comparisons (Fisher’s LSD tests).

In the baseline locomotion experiments, quarter turn data were analyzed using one-way ANOVA with CDPPB or fenobam treatment as the main factor.

## Results

### Elevated locomotion as a consequence of repeated METH treatment

**Figure 2.**
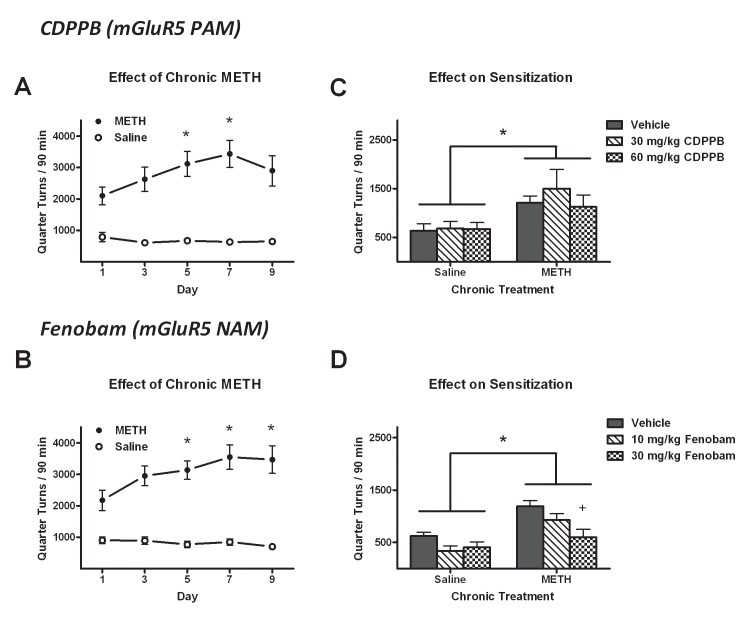
Effects of mGluR5 treatment by CDPPB (top row) or fenobam (bottom row) on locomotion and methamphetamine (METH) behavioral sensitization. In locomotor sessions prior to mGluR5-targeted treatment ( **A-B**), rats were chronically given 1 mg/kg METH (filled circles) or saline (open circles). In both the CDPPB ( **A**) and fenobam ( **B**) experiments, the reported quarter turns progressively increased above first-day levels in the METH-exposed groups. * *P* < 0.05 different from Day 1 levels. In the subsequent test using 0.5 mg/kg METH in all groups ( **C**), rats with a history of chronic METH exposure exhibited elevated locomotor behavior, but CDPPB pretreatment had no effect. In the fenobam experiment ( **D**), rats with a history of chronic METH exposure also exhibited elevated locomotor activity, and this behavioral sensitization was not affected by 10 mg/kg fenobam pretreatment. After 30 mg/kg fenobam treatment, the METH-sensitized locomotor response was reduced from the vehicle level. * *P* < 0.05 difference between METH history groups, regardless of mGluR5 ligand treatment. + *P* < 0.05 different from vehicle treated group with matching history of METH exposure. *PAM* stands for positive allosteric modulation, and *NAM* stands for negative allosteric modulation.

In the CDPPB experiment, rats treated with repeated METH injections exhibited progressively increasing amounts of quarter turns, as confirmed by a significant main effect of *METH history* ( *F*_1,164_ = 51.8, *p* < 0.0001) and a *day* × *METH history* interaction ( *F*_4,164_ = 3.4, *p* < 0.05). In these rats, locomotion was significantly elevated from Day 1 levels (2110 ± 284) on Day 5 (3117 ± 401, *p* < 0.05, Fisher’s LSD test) and Day 7 (3432 ± 433, *p* < 0.01), but not Day 9 ( Figure 2A and Table S1– Table S2). Similarly, in the fenobam experiment, repeated injections of METH but not saline resulted in elevated quarter turns, as confirmed by significant main effects of *day* ( *F*_4,172_ = 4.1, *p* < 0.005) and *METH history* ( *F*_1,172_ = 60.9, *p* < 0.0001) and a *day* × *METH history* interaction ( *F*_4,172_ = 6.0, *p* < 0.0005). In these rats, locomotion was significantly elevated from Day 1 levels (2175 ± 320) on Day 5 (3136 ± 297, *p* < 0.05, Fisher’s LSD test), Day 7 (3548 ± 388, *p* < 0.01) and Day 9 (3469 ± 438, *p* < 0.05, Figure 2B and Table S3– Table S4).

### Effect of mGluR5 modulation on locomotor sensitization to METH

In the CDPPB experiment, rats with a history of repeated METH treatments exhibited a greater number of quarter turns following a probe injection of 0.5 mg/kg METH, evidence of locomotor sensitization ( Figure 2C and Table S5– Table S6). This elevated response to METH was not attenuated by CDPPB pretreatment, as shown by the existence of a main effect of *METH history* ( *F*_1,37_ = 10.7, *p* < 0.005) but no other main effects or interactions.

In the fenobam experiment, rats with a history of repeated METH treatments also exhibited elevated quarter turns following the 0.5 mg/kg METH probe ( Figure 2D and Table S7– Table S8). Pretreatment with fenobam attenuated the locomotor response to METH, regardless of METH exposure history, as revealed by the presence of main effects of *METH history* ( *F*_1,39_ = 20.1, *p* < 0.001) and *treatment* ( *F*_2,39_ = 6.7, *p* < 0.005), but no *METH history × treatment* interaction. However, pretreatment with the large dose of fenobam (30 mg/kg) resulted in significantly reduced METH-induced locomotion in rats with a history of chronic 1 mg/kg METH injections (0 mg/kg fenobam: 1192 ± 105 quarter turns vs. 30 mg/kg fenobam: 597 ± 150 quarter turns, *p* < 0.01, two-sample *t*-test), and produced a trend toward a significant reduction in rats with a history of saline injections (0 mg/kg fenobam: 622 ± 493 quarter turns vs. 30 mg/kg fenobam: 405 ± 106 quarter turns, P = 0.08).

### Effect of mGluR5 modulation on baseline locomotion

**Figure 3.**
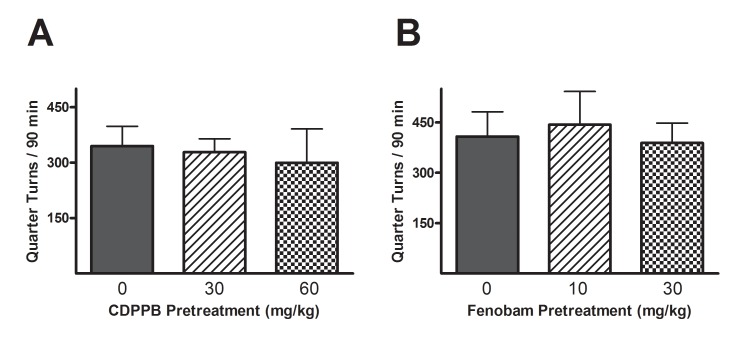
Effects of mGluR5 treatment on baseline locomotion in previously drug-naïve rats. CDPPB ( **A**) or fenobam ( **B**) was injected 30 min prior to locomotor testing. No significant effects were reported from the quarter turns collected over 90 min sessions.

All of the tested doses of CDPPB and fenobam had negligible effects on baseline locomotion, measured 30 min after time of injection. Both the 60 mg/kg dose of CDPPB (300 ± 92 quarter turns, vs. 345 ± 43 for the vehicle) and the 30 mg/kg dose of fenobam (389 ± 59 quarter turns, vs. 407 ± 74 for the vehicle) produced slightly attenuated locomotor responses, but no significant effects were revealed by ANOVA in either experiment ( Figure 3 and Table S9– Table S10).

## Discussion

As expected, rats repeatedly injected with 1 mg/kg METH exhibited greater locomotor activity than the saline-treated rats, and demonstrated more activity during the latter sessions than the initial session. Treatment with CDPPB did not significantly alter METH-induced rotational locomotion, and treatment with fenobam only significantly reduced rotational locomotion at its highest dose (30 mg/kg). Neither CDPPB nor fenobam significantly attenuated the baseline locomotor activity of drug-naïve animals, although the small effect found for 30 mg/kg fenobam in that experiment ( Figure 3B) could explain the moderate reduction of quarter turns exhibited by METH-challenged rats ( Figure 2D) as a non-specific phenomenon. Thus, locomotor effects of mGluR5 modulation were largely absent at the dose ranges that have been shown in earlier studies to reduce operant behavior motivated by METH or cocaine training ^7, 15, 16, 18, 19^.

These largely negative findings indicate that the maintenance of behavioral sensitization is likely mediated by neurobiological substrates other than mGluR5. These data are also in agreement with previous observations that mGluR5 function does not appear critical for the expression of locomotor sensitization to cocaine ^14, 20^, and extends them to include METH sensitization. Furthermore, the contribution of mGluR5 to initial locomotor responses to injected psychostimulants ^13^ appears to be replaced by other neurochemical substrates with chronic drug exposure.

While mGluR5 is an important therapeutic target in researching treatments for addiction to psychostimulants as well as other abused substances, there is building evidence that the role of this receptor in drug-related behaviors changes with increasing exposure. A recent study of rats chronically exposed to METH sufficient to induce measurable conditioned place preference found a reduction of surface expression of mGluR5 in the medial prefrontal cortex ^21^, an area known to contribute to the expression of behavioral sensitization ^4^. The current findings using the behavioral sensitization model therefore suggest that the changes in the degree to which mGluR5 mediates drug-stimulated and drug-conditioned behavior previously shown to occur with chronic cocaine exposure might also take place in rats with a history of chronic METH exposure. The possibility of the changing roles among the various mGluR subfamilies as a result of drug exposure merits further studies utilizing animal models of METH-induced activity and motivated behavior.

## Figures and Tables

**Table S1.** CDPPB experiment – locomotor response (total quarter turns over 90 min) after chronic METH treatments. In locomotor sessions prior to mGluR5-targeted treatment, rats were chronically given 1 mg/kg METH i.p. In this experiment, the reported quarter turns progressively increased above first-day levels.

Rat	Day of treatment (1 mg/kg METH)				
	1	3	5	7	9
**203**	2419	2269	3200	4701	1648
**205**	3840	3197	2640	6428	1867
**213**	2436	1520	3379	1243	2273
**234**	585	990	913	950	577
**238**	2119	1539	1046	2845	1151
**242**	1487	1825	1215	1412	1606
**244**	987	1063	3221	3230	1475
**201**	2907	2145	3695	5875	4264
**207**	1454	1568	3963	3442	2566
**211**	3581	2512	3086	3152	5037
**215**	1534	1727	3699	1804	1655
**232**	726	1229	1567	1737	1492
**236**	3436	7602	6724	7647	7239
**246**	2275	2439	6851	5386	4959
**248**	818	2449	1101	1434	2336
**253**	1016	1599	1306	1097	3678
**254**	415	3854	1492	4504	2005
**255**	4608	5091	3499	3836	3150
**256**	1672	1794	5353	4008	9378
**257**	1160	2158	5724	1625	1425
**258**	4639	6600	1770	5712	1024

**Table S2.** CDPPB experiment – locomotor response (total quarter turns over 90 min) after saline treatments. In locomotor sessions prior to mGluR5-targeted treatment, rats were chronically given 1 ml/kg saline i.p. The reported quarter turns did not significantly change from first-day levels.

Rat	Day of treatment (saline)				
	1	3	5	7	9
**202**	397	248	181	301	359
**206**	2964	247	1240	969	1621
**214**	342	408	1202	539	557
**235**	644	1205	750	858	653
**237**	668	919	863	983	675
**241**	295	516	890	634	646
**212**	423	607	322	442	289
**243**	420	557	331	449	683
**204**	448	321	435	367	288
**208**	923	940	730	855	1098
**216**	2078	1246	1651	960	1563
**231**	653	895	711	604	494
**233**	1265	640	803	917	612
**245**	1488	1151	817	820	1138
**247**	477	549	723	1160	885
**251**	74	178	381	214	424
**252**	67	26	77	124	128
**271**	316	797	454	391	298
**272**	202	202	190	226	136
**275**	1288	495	642	1063	495
**263**	959	681	941	576	681
**264**	922	490	421	347	445

**Table S3.** Fenobam experiment – locomotor response (total quarter turns over 90 min) after chronic METH treatments. In locomotor sessions prior to mGluR5-targeted treatment, rats were chronically given 1 mg/kg METH i.p. In this experiment, the reported quarter turns progressively increased above first-day levels.

Rat	Day of treatment (1 mg/kg METH)				
	1	3	5	7	9
**362**	315	1314	1818	1068	966
**364**	1691	1869	4040	3447	2381
**366**	3813	2074	3556	6491	7163
**368**	1261	2087	926	1961	2489
**377**	1888	3952	4491	3738	3905
**383**	1547	1065	3203	3511	2747
**385**	1989	1586	2476	3679	2865
**387**	1214	1960	536	1807	963
**352**	1983	1325	1693	1853	1865
**354**	2966	2963	4444	4726	5932
**356**	7984	5835	6043	6727	7125
**358**	1798	4432	3827	7331	6979
**371**	2167	2344	2538	2110	3273
**373**	2342	3220	1545	2069	2442
**375**	1796	3876	2117	3638	2653
**381**	1863	2059	3483	3319	3158
**313**	676	3157	2552	2467	5972
**314**	1868	5270	5345	2352	5141
**315**	3195	2660	3308	6766	951
**316**	1600	6267	3301	3516	3549
**317**	1741	3105	3223	1767	717
**318**	2154	2530	4528	3704	3091

**Table S4.** Fenobam experiment – locomotor response (total quarter turns over 90 min) after saline treatments. In locomotor sessions prior to mGluR5-targeted treatment, rats were chronically given 1 ml/kg saline i.p. The reported quarter turns did not significantly change from first-day levels.

Rat	Day of treatment (1 mg/kg saline)				
	1	3	5	7	9
**351**	979	1042	670	763	727
**357**	2092	2047	1343	1656	1664
**361**	418	369	348	387	433
**367**	1309	1444	1751	1440	1480
**372**	345	244	486	430	359
**374**	1120	1177	847	1412	1195
**384**	1307	613	878	598	730
**386**	1216	1368	939	1246	633
**353**	852	701	466	528	636
**355**	452	452	320	1445	1010
**363**	735	1092	1185	1084	733
**365**	1308	2251	2095	1649	1018
**376**	1406	748	1147	1024	1078
**378**	1146	762	816	948	599
**382**	540	191	393	438	567
**388**	1338	1233	970	1146	678
**311**	225	378	219	390	362
**312**	192	255	152	297	161
**323**	959	1028	941	576	681
**324**	922	490	421	347	445
**331**	316	797	454	391	298
**332**	202	202	190	226	136
**335**	1288	1623	642	1063	495

**Table S5.** CDPPB (0, 30, 60 mg/kg) effects on METH locomotor response (total quarter turns over 90 min) – rats with histories of saline injections. In the Day 16 tests using 0.5 mg/kg METH in all groups, rats with a history of chronic saline injections exhibited elevated locomotor behavior, but CDPPB pretreatment had no effect.

Rat	CDPPB	Quarter turns
**202**	0	910
**206**	0	215
**214**	0	363
**235**	0	952
**237**	0	1001
**241**	0	871
**212**	0	135
**243**	30	1495
**204**	30	885
**208**	30	129
**216**	30	692
**231**	30	281
**233**	30	744
**245**	30	683
**247**	30	539
**251**	60	1117
**252**	60	358
**271**	60	668
**272**	60	127
**275**	60	1113
**263**	60	681
**264**	60	622

**Table S6.** CDPPB effects on METH locomotor response (total quarter turns over 90 min) – rats with histories of METH injections. In the Day 16 tests using 0.5 mg/kg METH in all groups, rats with a history of chronic METH exposure exhibited elevated locomotor behavior, but CDPPB pretreatment had no effect.

Rat	CDPPB	Quarter turns
**203**	0	1425
**205**	0	1767
**213**	0	1112
**234**	0	933
**238**	0	1100
**242**	0	653
**244**	0	1475
**201**	30	542
**207**	30	1674
**211**	30	1325
**215**	30	1701
**232**	30	904
**236**	30	1858
**246**	30	3808
**248**	30	210
**253**	60	345
**254**	60	397
**255**	60	1675
**256**	60	1414
**257**	60	1252
**258**	60	1662

**Table S7.** Fenobam (0, 10, 30 mg/kg) effects on METH locomotor response (total quarter turns over 90 min) – history of saline injections. In the Day 16 tests using 0.5 mg/kg METH in all groups, rats with a history of chronic saline injections exhibited elevated locomotor behavior, but fenobam pretreatment had no effect.

Rat	Fenobam	Quarter turns
**351**	0	257
**357**	0	770
**361**	0	661
**367**	0	909
**372**	0	449
**374**	0	587
**384**	0	693
**386**	0	656
**353**	10	748
**355**	10	181
**363**	10	394
**365**	10	725
**376**	10	298
**378**	10	910
**382**	10	480
**388**	10	207
**311**	30	315
**312**	30	101
**323**	30	274
**324**	30	219
**331**	30	955
**332**	30	465
**335**	30	508

**Table S8.** Fenobam (0, 10, 30 mg/kg) effects on METH locomotor response – history of METH injections. In the Day 16 tests using 0.5 mg/kg METH in all groups, rats with a history of chronic METH exposure exhibited elevated locomotor behavior, and 30 mg/kg but not 10 mg/kg fenobam resulted in reduced quarter turns relative to vehicle-pretreated animals.

Rat	Fenobam	Quarter turns
**362**	0	1551
**364**	0	1190
**366**	0	1111
**368**	0	611
**377**	0	1509
**383**	0	1354
**385**	0	1050
**387**	0	1162
**352**	10	929
**354**	10	1263
**356**	10	1084
**358**	10	1391
**371**	10	861
**373**	10	614
**375**	10	281
**381**	10	1009
**313**	30	275
**314**	30	927
**315**	30	419
**316**	30	619
**317**	30	218
**318**	30	1129

**Table S9.** Locomotor response (total quarter turns over 90 min) to CDPPB (0, 30, 60 mg/kg).

Rat	CDPPB	Quarter turns
**101**	0	304
**104**	0	171
**107**	0	490
**110**	0	353
**113**	0	407
**102**	30	353
**105**	30	401
**108**	30	198
**111**	30	384
**114**	30	307
**103**	60	650
**106**	60	120
**109**	60	245
**112**	60	199
**115**	60	285

**Table S10.** Locomotor response (total quarter turns over 90 min) to Fenobam (0, 10, 30 mg/kg).

Rat	Fenobam	Quarter turns
**403**	0	365
**406**	0	577
**409**	0	584
**412**	0	226
**415**	0	286
**401**	10	317
**404**	10	468
**407**	10	339
**410**	10	274
**413**	10	817
**402**	30	478
**405**	30	465
**408**	30	274
**411**	30	219
**414**	30	508
