# Supplementary material for: Positive or negative allosteric modulation of metabotropic glutamate receptor 5 (mGluR5) does not alter expression of behavioral sensitization to methamphetamine
Source: F1000Res. 2013 Mar 12;2:84. [Version 1] doi: 10.12688/f1000research.2-84.v1 (PMC3814922; doi:10.12688/f1000research.2-84.v1)
Supplement: Peer review history

# Peer review: Referee response for version 1

**Published:** 2013-06-06

**Authors:** Sharon Rosenzweig-Lipson

**Affiliations:** 1 Pharmacology & Physiology Faculty, IVS Pharma Consulting LLC, East Brunswick, NJ, USA

The present studies investigated the effects of positive and negative allosteric modulation of mGluR5 receptors on methamphetamine sensitization. The authors conclude that “Positive or negative allosteric modulation of metabotropic glutamate receptor 5 (mGluR5) does not alter expression of behavioral sensitization to methamphetamine”. While the data, in part, support those conclusions; the presence of an effect of 30 mg/kg fenobam on methamphetamine sensitization suggests at least some role of mGlur5 NAM activity. Evaluation of an additional NAM or a higher dose of fenobam would allow for a firmer conclusion on this point.

I have read this submission. I believe that I have an appropriate level of expertise to confirm that it is of an acceptable scientific standard, however I have significant reservations, as outlined above.

# Peer review: Referee response for version 1

**Published:** 2013-05-14

**Authors:** Bianca Jupp

**Affiliations:** 1 Behavioural and Clinical Neuroscience Institute, University of Cambridge, Cambridge, UK

The publication by Kufahl and colleagues presents an investigation into the effect of positive and negative allosteric modulators of mGluR5 on the expression of locomotor sensitization to the psychostimulant methamphetamine, the results of which apparently support previous data regarding a lack of involvement of this receptor in the expression of sensitized locomotion. While the study is well designed, a critical component of the results was omitted making the interpretation of the current data impossible, and severely undermines the author’s conclusions. 

Specifically, while the authors methodologically included a saline challenge when assessing the expression of sensitization, they failed to report these results. Without this it is not possible to determine if indeed the increase in locomotor activity observed in the METH pre-treatment group is due to expression of conditioned hyperactivity or locomotor sensitization. I suspect it may be the former due to the apparently reduced locomotor activity (approx 1200) observed during this challenge session even when compared to acute METH (approx 2000). Usually expression of locomotor sensitization is much greater than the final conditioning session. It is therefore unreasonable for the authors to conclude that PAM or NAM of mGluR5 has no effect on expression of sensitization as it is not even clear if the animals are expressing sensitized behaviour. Inclusion of the saline challenge data will clarify this point. 

Have the authors considered using a longer ‘waiting’ period between development and testing expression? A recent study by Timmer and Steketee, 2012 found that intra-prefrontal cortex injections of the mGluR5 PAM MTEP reduced the expression of locomotor sensitization to cocaine following 21 days but not 7 days. The authors should include this in the discussion of their results.

I have read this submission. I believe that I have an appropriate level of expertise to confirm that it is of an acceptable scientific standard, however I have significant reservations, as outlined above.

# Peer review: Referee response for version 1

**Published:** 2013-03-18

**Authors:** David Triggle

**Affiliations:** 1 School of Pharmacy and Pharmaceutical Sciences, SUNY at Buffalo, Buffalo, NY, USA

Although this is a report of primarily negative findings it is not without value and should be published. The premise of the research is reasonable, the methods appropriate and the conclusions appropriate and not overreaching.  Essentially, the workers have demonstrated through behavioural studies in rats that allosteric modulation – either positive or negative – of the metabotropic glutamate receptor 5 does not modify methamphetamine-induced behavioural sensitization. This adds to our knowledge of the effects of methamphetamine in its abuse.

I have read this submission. I believe that I have an appropriate level of expertise to confirm that it is of an acceptable scientific standard.
